# Challenges of *Dermanyssus gallinae* in Poultry: Biological Insights, Economic Impact and Management Strategies

**DOI:** 10.3390/insects16010089

**Published:** 2025-01-16

**Authors:** Péter Sárkány, Zoltán Bagi, Ágnes Süli, Szilvia Kusza

**Affiliations:** 1 Centre for Agricultural Genomics and Biotechnology, University of Debrecen, H-4032 Debrecen, Hungary; sarkany.peter.deb@gmail.com (P.S.); bagiz@agr.unideb.hu (Z.B.); 2 Institute of Animal Sciences and Wildlife Management, University of Szeged, H-6800 Hódmezővásárhely, Hungary; suli.agnes@szte.hu

**Keywords:** acaricide resistance, alternative treatments, *Dermanyssus gallinae*, economic impact, integrated pest management, genomics

## Abstract

Bird mites are parasites that feed on both wild and domesticated bird species, causing severe degradation in avian welfare. The chicken mite, *Dermanyssus gallinae* in particular, is a widespread ectoparasite in poultry, responsible for several challenges faced by the poultry industry, including poor animal health, which causes significant economic losses. This review, based on our current knowledge, aims to provide a comprehensive insight into the biology and distribution of these mites, as well as their impact on poultry health and production. It explores the most prevalent mites in avian species, with a focus on *D. gallinae*, and examines the different psychological and physiological alterations observed in infected stocks, such as decreased egg production, weight loss, and an increased susceptibility to diseases. This review will also cover existing control strategies, including chemical, biological, and environmental approaches, with attention to the growing concern around pesticide resistance. Additionally, it delves into genetic research conducted on these mites, primarily focusing on phylogenetic studies, which have provided insights into their evolutionary relationships and potential vulnerabilities. By compiling existing studies, this article underscores the urgent need for effective and sustainable countermeasures, as well as further genetic research to mitigate the substantial impact of *D. gallinae* on the poultry sector.

## Simple Summary

*Dermanyssus gallinae,* or poultry red mite, is a blood-sucking opportunistic parasite that is the leading cause of losses in the poultry sector, especially in Europe. With their ability to proliferate rapidly, to utilise human activity as a form of transmission, and to starve for long periods of time, these mites have achieved high infestation rates in many countries, both inside and outside of Europe. Red mites are a huge cause for concern in terms of animal welfare, causing anaemia, pain, intense itching, and many psychological effects due to the stressful environment they generate. This, combined with significant economic losses, has necessitated the development of many different control strategies. Methods for mite infestation control include synthetic and natural pesticides, repellents, the use of natural predators and microorganisms, physical control methods, and integrated pest management strategies. This review delves into the biology, distribution, economic impact, health and safety concerns, management strategies, challenges, and available genetic and phylogenetic research surrounding *Dermanyssus gallinae*, while also pointing out potentially relevant topics upon which future studies could be based.

## 1. Introduction

*Dermanyssus gallinae,* or poultry red mite (PRM), is a hematophagous ectoparasite feeding on a large variety of avian species, including but not limited to farmed pheasants, turkeys, ducks, and even domesticated pigeons [1]. However, what this mite is most notorious for is its negative effect on poultry welfare and therefore its significant impact on the poultry industry. PRM-infested hosts show physiological symptoms such as anaemic symptoms and painful and itchy bitemarks, resulting in stress-related psychological effects such as aggression, restlessness, behavioural changes, and, in serious cases, even cannibalism [2]. *D. gallinae* infestation is a worldwide concern that is responsible for significant economic losses in the poultry sector. With losses adding up to the hundred-million-euro range in the European Union alone, finding effective control measures has become the number one priority for both researchers and farmers alike [3]. However, finding these practices has not been an easy task, with each strategy having its own drawbacks, but also because of *D. gallinae*’s incredible ability to adapt, forcing proven countermeasures to become obsolete. Nevertheless, there has been a large number of publications regarding *Dermanyssus gallinae* and its control strategies, which served as the basis of this paper. The main objective of this review is therefore simple: to provide comprehensive general knowledge. By compiling key elements across *D. gallinae*’s biology, distribution, impact on animal welfare, control strategies, and challenges, this review aims to provide a broad yet detailed understanding accessible to both specialists and non-specialists, while also offering directional guidance for future research topics.

## 2. Biological Insights into *Dermanyssus gallinae*

**Figure 1:**
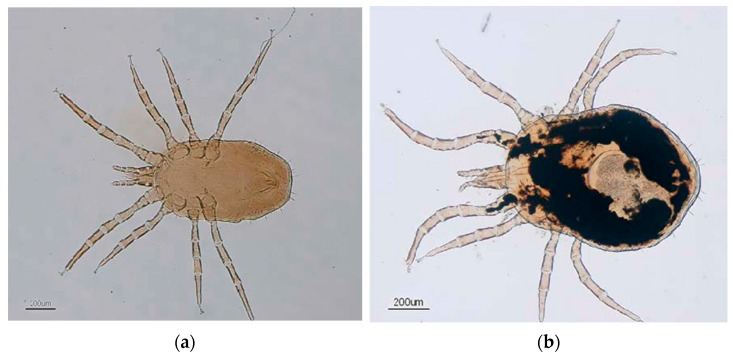
Images of *Dermanyssus gallinae*: (**a**) male mite; (**b**) female mite (with egg) [7].

*Dermanyssus gallinae* (Figure 1), a blood-sucking ectoparasite of birds, is the most economically and medically significant member of the *Dermanyssus* genus (Arachnida: Mesostigmata: Dermanyssidae) and includes species like *Dermanyssus prognephilus* (a common parasite of purple martin chicks), *Dermanyssus hirundinis* (a common parasite of house wren offspring), and *Dermanyssus americanus* (found in North American bird species) [4,5]. Though around 24 species are recognised, taxonomic challenges like cryptic species and geographical variation persist, necessitating further research to refine species classification [5,6].

Morphologically, *Dermanyssus* species fall into the hirsutus group (distinct traits and host associations) or the gallinae group (variable traits, less distinguishable) [5,8]. Some studies, however, question the validity of this division [9]. *D. gallinae* is small (1–1.5 mm), with a chitin exoskeleton that provides durability and flexibility for engorgement. Sensory setae, located on the legs (four pairs), dorsal shield, genitoventral shield, and anal shield of the mite’s body, aid in host detection through heat, vibration, and CO_2_ sensing [10,11,12,13]. Specialised legs with retractable pulvilli enable mobility on slippery surfaces and olfactory sensing, making the mite highly adaptive [14,15]. These traits provide potential avenues for control research.

**Figure 2:**
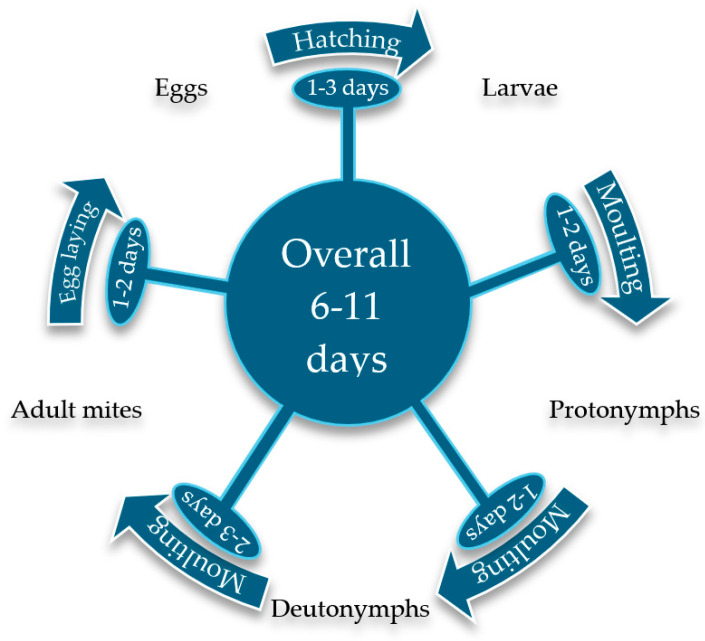
The lifecycle of *Dermanyssus gallinae*.

The mite’s life cycle spans 1–2 weeks (Figure 2), starting with an engorged female laying 1–9 eggs, which hatch into larvae in 1–3 days. The larvae moult into mobile protonymphs and, after feeding, into deutonymphs before reaching adulthood. Females can lay 30–200 eggs in their lifetime [10,16,17]. Optimal development occurs at 30 °C, while heat stress at 35 °C reduces reproduction and development speed, suggesting its potential as a control strategy [18].

*D. gallinae* is a nocturnal ectoparasite, feeding on its host at night and hiding in cracks and crevices during the day. It locates hosts through sensory cues like CO_2_, kairomones (originating from host), and pheromones (originating from fed mites) [19]. Females feed every 2–4 days, while males feed less often [17]. Remarkably resilient, *D. gallinae* can survive over a year without feeding; therefore, infested facilities may require a 2-year break to ensure eradication, highlighting the mite’s persistence and the challenges it poses to poultry management [20].

## 3. Distribution and Prevalence

**Table 1:** Prevalence of *Dermanyssus gallinae* in different European and non-European countries.

Country	Prevalence (%)	Reference
Portugal	95%	[21]
Belgium	94%	[22]
South Korea	90%	[23]
United Kingdom	87%	[22]
Italy	83%	[22]
Greece	75%	[24]
Turkey ^1^	72%	[25]
France	67%	[22]
Romania	64%	[26]
Tunisia	34%	[27]

^1^ Data from Canakkale province.

Poultry red mite infestations affect many countries from all over the world, with the most problematic countries being located in South America, the Middle East, Asia, and Europe [28]. The overall PRM prevalences in these countries vary from each other (Table 1), possibly due to their climate and different production systems. Just to give a few examples, prevalence is estimated at 34% in north-east Tunisia, 75% in Greece, and even 90% in South Korea [23,24,27]. Infestation rates as high as 80–90% are not rare; in fact, they have been observed in many European countries, such as the United Kingdom, the Netherlands, Poland, Serbia, Italy, Morocco, Spain, and Portugal [21,29,30]. Seeing how many European countries have such high infection rates, one could wonder why this may be. Part of the answer lies within the 1999/74/EC directive, which banned the use of traditional cage systems in poultry farming, starting in 2012. This legislation aimed to improve poultry welfare and required farmers to abandon the use of cages and to adopt new “cage-free” technologies, providing animals with the ability to move more freely while still in a confined space. With this change, however, chicken houses became much more complex, making the cleaning and sanitation process more difficult. These environmental changes were in favour of *Dermanyssus gallinae*, making living, proliferation, and feeding much easier for the mite [28,31]. Before the effective ban on traditional cages in Europe, they had been in use since the 1950s and were generally recognised (alongside the emergence of modern management practices) to have a mitigating effect on mite infestation; after the ban, however, mite infestation rates became significantly higher [32,33]. Increasing numbers of PRM-associated problems were also observed in Switzerland, where the use of traditional cages was banned in 1992, long before the EU directive, and also in the Netherlands where the initial tests of alternative housing systems were being performed at the time [32,33,34]. Europe’s incidence of the red poultry mite became a warning to many countries, and as a result, the majority of egg production from large-scale producers continues to be sourced from hens kept in cages [35,36]. However, growing consumer demand for higher welfare standards is seemingly causing a shift towards non-cage systems in several non-EU countries, such as Canada, Australia, New Zealand, and even the US, where PRM infestations are not as serious as in Europe. However, with a significant fraction of the poultry sector adopting alternative housing systems, a resurgence of *D. gallinae* is to be expected [33,36,37,38].

It is important to mention that these ectoparasites are not exclusively chicken mites. On the contrary, they can be found to feed on a number of other avian species, even wild birds. A recent study analysing blood and faecal samples from multiple captive bird species showed that *Dermanyssus gallinae* was present in turkeys and chukars [39]. As mentioned before, PRM lives in small cracks and crevices, hiding from predators. This means that for a bird to be infected by *D. gallinae*, it must live near sites where these hiding places are in abundance, for example, barns, abandoned buildings, lofts, etc. For this reason, among wild birds, infestation rates are the highest in wild pigeons. *Dermanyssus gallinae* can also naturally reside in birds’ nests. For example, a 2020 research study from Bulgaria showed that *D. gallinae* was present in almost half (44.96%) of the investigated bird nests (belonging to semi-collared flycatcher (*Ficedula semitorquata*), great tit (*Parus major*), and Eurasian blue tit (*Cyanistes caeruleus*)) [40]. This goes to show that *Dermanyssus gallinae* distribution is not limited to poultry farms, making exact distributional data generation difficult.

However, by understanding distinct factors influencing its distribution, we can eliminate habitats which are favourable to poultry red mites. We have already mentioned the need for hiding places, which are quite essential for *D. gallinae* to thrive. Another essential factor is darkness, which is necessary for the mite to reach an optimal proliferation rate [41]. However, temperature and climate are of greater significance. Poultry red mites are extremely sensitive to temperature changes, being most productive at around 28–30 °C. While extreme cold is acceptable for colony maintenance in laboratories [42], prolonged cold exposure decreases the efficiency of the mite’s reproduction. The same is true for extreme heat (>35 °C), making sites with such conditions virtually uninhabitable for PRM [18,43]. The mite’s temperature sensitivity is further supported by a study conducted around Cluj, Romania, where the maximal infestation rate was during the summer season (~87%), while the lowest was during the winter season (~38%) [26]. But the main factor in *Dermanyssus gallinae*’s distribution might be human activity. As poultry farms are largely affected by poultry red mites (especially in Europe), the logistical network between farms and other highly frequented hubs is the perfect mode of distribution, all of which can occur while transporting animals/farm equipment or supplying pullets [44].

## 4. Impact on Poultry Health

*Dermanyssus gallinae* infestation has a significant negative impact on the health of birds, causing a number of physiological, psychological, and behavioural changes. Mites usually feed on hens every 2–4 days, and with an average amount of 25,000–50,000 mites per bird (or, in extreme cases, 500,000 mites per bird) hens can lose up to 3% of their total blood volume, which eventually leads to regenerative anaemia (i.e., blood loss quicker than erythropoiesis) or, in extreme cases, severe anaemia [22,31,45]. Bloodwork analysis conducted on infected birds showed significant decreases in the number of red blood cells and platelets and slight decreases in haemoglobin levels [31,46]. A major drop in γ-globulin levels and an increase in β-globulin levels were also observed, indicating immunosuppression. There was also an increase in corticosterone levels, indicating somatic stress, and elevated adrenaline levels, which may suggest heightened physiological responses to multiple stressors, including those potentially caused by mite infestations [46,47]. Elevated liver function values also suggest that poultry red mite saliva may have a hepatotoxic effect on the birds [47]. Mite saliva also causes itching and irritation, and with thousands of bites inflicted upon them, hens are under considerable amount of stress. This leads to the birds being irritated, restless, or even outright aggressive. Itching and skin irritation also induce behavioural changes such as feather-pecking (gentle or severe), body shaking, head scratching, preening, and even cannibalistic behaviour [48,49,50]. Infested birds also lose their appetite, which reduces their weight gain and hinders their growth [50]. PRM infestation also causes decreased laying performance (approx. 15–20%) by activating the hypothalamic–pituitary–adrenocortex axis (somatic stress), which releases hormones that supress the axis responsible for egg laying [45,46], while egg quality also decreases due to shell thinning [51]. Infected, anxious, stressed, and immunosuppressed hens are also more likely to catch a variety of diseases, especially if we take into consideration that *Dermanyssus gallinae* is a known vector of avian pathogens, such as *Avipox virus*, *Marek’s disease virus*, *Erysipelothrix rhusiopathiae*, *Salmonella enterica*, *Escherichia coli*, *Staphylococcus* species, and many more [52,53,54,55]. So, it is because of the above-mentioned health related reasons that many infested flocks experience a higher mortality rate. These torturous and unacceptable conditions definitely underline the need for effective control strategies.

## 5. Economic Implications

**Figure 3:**
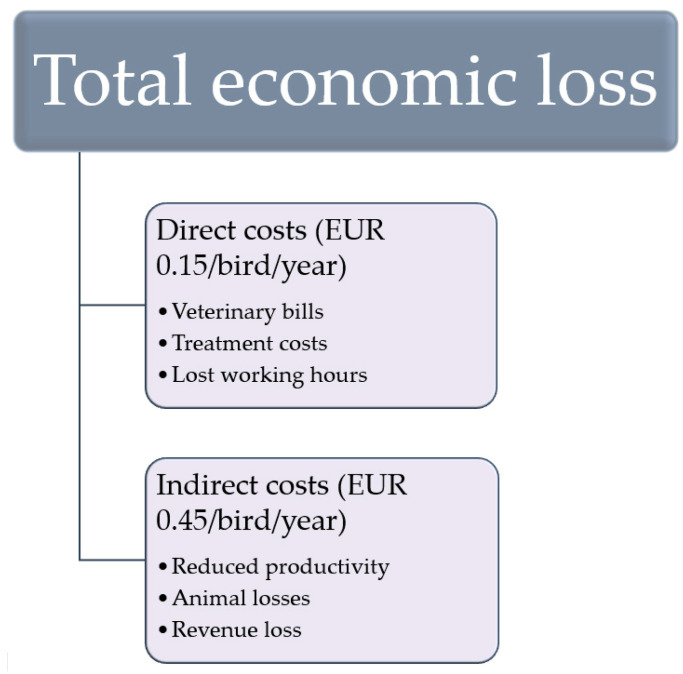
A comparison between the types of losses caused by *Dermanyssus gallinae*.

With its huge prevalence in poultry farming, *Dermanyssus gallinae’s* economic impact is quite substantial. How substantial it is exactly is, however, a mystery as of writing this paper. Economic losses can be broken down into two groups: direct costs and indirect costs (Figure 3). Under direct costs fall a number of expenditures which directly correlate to PRM infestation; these include expenses like veterinary bills, treatments costs, and lost working hours. Indirect costs refer to losses suffered as a consequence of the infestation, such as reduced productivity, decreased revenue, and animal losses [29].

A highly referenced 2005 study estimated the direct costs and indirect costs to be EUR 0.14/bird/year and EUR 0.29/bird/year, respectively (considering moderate infestation), and the overall losses to be ~ EUR 130 million in Europe alone [31,56]. However, this figure is surely outdated, as almost two decades have passed since then, bringing with them changes in costs, animal numbers, infection rates, and legislative measures, all of which influence the expenses quite a bit. The same author estimated in 2017 that these expenses had risen to EUR 0.15 for direct and EUR 0.45 for indirect costs in the Netherlands, meaning EUR 0.60 in losses per bird per year and EUR 231 million in overall damage in Europe [3,56], which is a much more acceptable number, but since then, another 7 years have passed. According to the European Commission, in 2021, there were 376 million laying hens in the EU [57], and with 80–90% infestation rates in many European countries, losses are expected to be even more than the 2017 estimate. However, with few to no economic assessments and outdated estimates, we cannot come to realise the true extent of losses inflicted by *Dermanyssus gallinae* in Europe, let alone globally, which at the moment appears to be an impossible undertaking. This lack of data reflects a broader challenge in pest management research, where there is often limited collaboration between biologists and economists. To address this gap, fostering interdisciplinary partnerships could help generate robust, well-accepted studies on the economic implications of pest infestations, thereby improving the design and evaluation of management strategies. Nevertheless, the European losses add up to hundreds of millions of euros, and with an estimated 4 billion infested layers worldwide [3], global losses may very well be in the billions.

## 6. Current Management Strategies

The above-mentioned animal health/welfare concerns and monetary implications gave rise to many control strategies that are in use today as a means to mitigate the damage caused by *Dermanyssus gallinae*. These control strategies can be categorised into two groups: conventional methods and alternative methods.

### 6.1. Conventional Control Strategies

Conventional methods focus on prevention through regular cleaning and sanitization, combined with commercially available synthetic acaricides [10]. These acaricides include organophosphates, organochlorines, pyrethroids, carbamates, macrocyclic lactones, endectocides, amitraz, formadin, isoxazolines, and dichloro-diphenyl trichloroethane (DDT) [10,28,31,58]. These compounds primarily target neurotransmission, causing paralysis, which prevents feeding and ultimately leads to death [13]. Despite their widespread use, acaricides face significant limitations, including efficacy issues, licencing restrictions, safety concerns, and emerging resistance, driving the need for alternative approaches.

### 6.2. Alternative Control Strategies

Finding alternative acaricides has been the goal of research papers for many years now, with varying degrees of success. Thuringiensin, the exotoxin of *Bacillus thuringiensis*, is one of the proposed alternatives. It can damage the cuticle of *D. gallinae*, resulting in mobility loss [59]. However, thuringiensin possesses the ability to inhibit the biosynthesis of RNA-polymerase, rendering it toxic to almost all life-forms, including vertebrates, making its usage in the poultry industry questionable [2,58,60]. On the other hand, Spinosad, a natural compound originating from the fermentation of *Saccharopolyspora spinosa*, has proved to be effective against *D. gallinae* infestation and has been approved for commercial use since 2010 in many EU countries under the name Elector^®^ [2,61]. Lithium chloride could also possibly be an alternative acaricide; according to a recent study, lithium chloride—a lithium salt which has proved to be an effective control method for the *Varroa* mite (parasite of bees)—showed promising results when used against poultry red mite, but further studies are required to establish its relevance [62].

#### 6.2.1. Volatile Organic Compounds

Many research papers have reported the usefulness of volatile organic compounds (VOCs) against PRM, inclusing pheromones, kairomones, and VOCs in essential oils and plant extracts. Pheromones and kairomones act as natural attractants of *Dermanyssus gallinae*; therefore, when combined with known acaricides, they could prove useful in making mite traps based on the attract-and-kill principle [63]. Essential oils and plant extracts, on the other hand serve, as natural repellents or even acaricides, depending on their concentration. The essential oil of *Artemisia sieberi* (“the desert worm wood”) was proven to be an effective repellent of adult poultry mites [56,64], just as garlic, thyme, and lavender oils, which are also great acaricides [56,63]. Red Mite Avian (Lentypou+) is a liquid treatment containing thyme (*Thymus* spp.), burdock (*Arctium* spp.), and tansy (*Tanacetum vulgare*) extracts that can possibly render the host’s blood repulsive to mites, causing them to starve [2]. This could come as an advantage, as a 2008 study suggests that starving mites are also more susceptible to the toxic effects of essential oils and possibly standard synthetic acaricides as well [65]. Mite-Stop^®^ (Vichte, Belgium) is another commercially available acaricide that uses a plant-derived oil, more specifically neem oil. This product was proven to have an advantage in efficacy over ByeMite^®^ (Cuxhaven, Germany), a product using phoxim (a synthetic acaricide) as its active ingredient [2,66]. All this just goes to show the true potential of using plant-derivative products in the war against poultry red mites.

#### 6.2.2. Natural Predators and Entomopathogenic Microorganisms

Biological control of *Dermanyssus gallinae* by introducing natural predators or entomopathogenic microorganisms into its habitat is also a feasible strategy. There are a number of predators that have been identified as predators of *D. gallinae*, such as *Androlaelaps casalis*, *Hypoaspis aculeifer,* and *Hypoaspis miles*. These predatory mites, found in proximity to *D. gallinae* habitats (for example, nests) are known to consume poultry red mites; therefore, they are capable of thinning their numbers in infested areas. However, ensuring their field efficacy could prove difficult considering various environmental factors and the potential presence of alternative prey [2,67]. Entomopathogenic microorganisms such as fungi and bacteria cause infections which result in the death of the host. Entomopathogenic fungi directly penetrate the host’s body, after which their mycelium proliferates in the haemolymph, eventually killing their host. Such fungi are *Beauveria bassiana*, *Trichoderma album*, *Paecilomyces fumosoroseus*, *Metarhizium anisopliae*, and *Aspergillus oryzae*; these have been proven to be able to kill *D. gallinae* with varying efficacy. For example, *M. anisopliae* in combination with *B. bassiana* achieved a mortality of 61.7% in field conditions when used at a higher dosage, while *B. bassiana* in combination with *T. album* could kill up to 80% of treated mites within 10 days. These two latter fungi were also used in so-called fungus traps, with a much higher mortality rate of 80–100% in field conditions [28,68]. *B. bassiana* in itself has also demonstrated significant efficacy against PRM, with laboratory studies reporting mortality rates exceeding 70% using various application methods and field tests showing reductions in mite populations of up to 65–90% when implemented via autoinoculation devices or sprayed conidia suspensions [69,70].

Among entomopathogenic bacteria, the most notable is *Bacillus thuringiensis*, which acquires its lethality from biodegradable proteins, insecticidal crystals, exotoxins, and extracellular proteases. With an observed mortality rate between 60 and 80%, depending on the length of the application and the concentration used, *B. thuringiensis* could be a promising method for control [68].

#### 6.2.3. Physical Control Strategies

Apart from chemical and biological control measures, physical control could also play a key role in mitigating infestation. These measures include temperature control (the use of extreme temperatures in depopulated housing facilities, causing high mortality), intermittent lighting schedules (disrupting the nocturnal cycle of mites), and the use of inorganic substances, such as diatomaceous earth (DE), kaolin, and silicas. These fine powder-like substances mainly focus on dehydration via cuticle damage, while also relying on creating a stressful environment for the mites that prevents movement. The effectiveness of these strategies can be seen in a 2020 study [71], where DE in liquid preparation showed high efficacy in controlling Dermanyssus gallinae, achieving a 94.7% reduction in mite populations in field experiments when applied at 10% concentration alongside mechanical cleaning, presenting a viable alternative to chemical acaricides and aiding in resistance management [2,72,73].

The main goal of environment management practices is to limit the hiding places suitable for *D. gallinae* in order to decrease the risk of infestation. This means analysing and mapping potential places where mites could take shelter and terminating them. This might sound like an arduous undertaking; however, by making sure that there are no places for *D. gallinae* to establish itself, the risk of infestation could drop significantly. Sanitation might appear to be an obvious measure, yet it remains highly underrated, despite being the cornerstone of prevention [2].

#### 6.2.4. Integrated Pest Management

Integrated pest management is an up-and-coming new concept which basically incorporates all previously mentioned control methods. It is a widely used strategy in horticulture; however, its use in animal husbandry is still at its beginning stages but shows promising results [74,75]. Integrated pest management (or IPM)—being a combination of control strategies—has eight principles (or steps) that should be kept in mind when planning. These eight principles are as follows: 1. prevention, 2. use of monitoring and forecasting systems, 3. treatment planning based on results, 4. use of non-chemical methods, 5. use of selective synthetic acaricides, 6. reducing the use of acaricides, 7. addressing acaricide resistance causes, and, finally, 8. evaluating results [76]. By following these steps, mite infestation management could become more effective and sustainable while also becoming equally safer for animals, humans, and the environment. Just to illustrate its ingenuity, one popular topic in IPM research is the exploitation of the olfactory sensitivity of PRMs [77]. This can be achieved, for example, by creating traps which use the previously mentioned attract-and-kill principle, but instead of using synthetic acaricides, traps could combine natural attractants with natural acaricides, such as carvacrol (monoterpene phenol found in essential oils) or even cold atmospheric-pressure plasma (CAPP) [78,79]. By combining these alternative methods, traps can become more selective towards poultry red mites while also being less harmful to the environment.

#### 6.2.5. The Possible Use of Machine Learning

With the rapid development of technology, computer simulation, artificial intelligence (AI), deep learning (DL), and machine learning (ML) technologies have become more advanced and more available for use. Therefore, integrating them into our search for control strategies does not seem to be as far-fetched as it would have years ago. These tools could provide useful assistance in a number of time- and labour-intensive tasks. By simulating population dynamics, researchers would be able to test potential control methods in silico, selecting the most promising methods as the basis for their next research, saving precious time by doing so [80]. In a recent study by Wang et al., researchers used boosted regression tree (BRT) model, a machine learning algorithm, in trying to map the distribution and risk assessment of *Orientia tsutsugamushi* (a hematophagous mite) [81]. DL was also used in determining the infestation levels of *Varroa destructor*, a mite affecting the honeybee population. The traditional way of evaluating infestation levels is by counting the number of mites that have fallen onto a sticky board positioned under the colony. This is, however, a very time-consuming task. By feeding pictures of the boards into a deep learning algorithm, realistic results were archived, which could yet again save significant amounts of time [82]. This could probably be implemented into *D. gallinae* infestation evaluation by taking pictures of different traps (for example cardboard traps, like in free-range poultry farms [83]) and feeding them into small object detection software. ML could also be used to evaluate different animal behaviours [84]. In a 2023 study, researchers used on-animal sensors in combination with an ML algorithm to try and detect sheep that had been infected by *Haemonchus contortus* (Barber’s pole worm) [85], and while further research is needed, this form of detection may very well be a promising method. On-animal sensors have also been used to study how *Ornithonyssus sylviarum* (another poultry ectoparasite) alters chickens’ behaviour [86]. As mentioned before, *Dermanyssus gallinae*-infested hens exhibit a number of obvious behavioural changes. In the future, the use of on-animal sensors combined with deep learning could prove useful. By establishing behaviour baselines and with sensors being able to provide continuous data collection, DL algorithms could be used to differentiate between normal and abnormal behaviours, making the early detection of behavioural changes—and therefore the detection of infestation—more achievable. However, as of writing this paper, these are only theories, and further research is needed to determine the viability, effectiveness, and optimal practices involving this form of detection and the use of ML on *D. gallinae* in general.

## 7. Challenges and Emerging Issues

Despite various control strategies, *Dermanyssus gallinae* infestations remain widespread, presenting significant challenges. A key issue is the rapid development of resistance to synthetic acaricides. For example, phoxim was approved in 2010, but resistant strains were already suspected in Poland by 2011, with widespread resistance detected by 2015 [87,88]. Throughout the years, a large number of acaricides were used against PRM infestation; however, resistance to all of them has been described throughout the world [89]. What makes this situation even more difficult is the fact that many of the previously used organophosphates, carbamates, and synthetic pyrethroids have since been withdrawn from the market, or their use has even been banned in Europe; therefore, available acaricides are limited not only by resistant species but legislative measures as well. As a result, farmers often resort to unapproved chemicals like λ-cyhalothrin, which also lose efficacy over time [90,91]. Resistant strains are also not region-locked, meaning that acaricide resistance affects several countries. In 2019, it was shown that pyrethroid resistance affects most European countries, and on account of human activity, acaricide resistance also affects countries outside of Europe [92]. With limited approved acaricides available, some (e.g., fluralaner) are expensive and require veterinary prescriptions, which complicate their accessibility [93]. While acaricide resistance usually originates from the improper, subtherapeutic dosing of chemicals, over-usage carries other types of danger, namely acaricide residues found in the tissue of chickens, as well as their eggs. In most cases, human exposure to pesticides happens because of the consumption of contaminated food, which poses several health risks [94].

*Dermanyssus gallinae* is also a direct threat to human health, causing dermanyssosis, an occupational hazard for poultry workers. Symptoms include painful bites (1–3 mm) and intense itching, often worsened by scratching. Diagnosis is challenging due to the nonspecific nature of these symptoms. Cases of dermanyssosis have also been reported in urban settings, often linked to infested pigeons [95,96]. Additionally, *D. gallinae* can transmit avian pathogens, raising concerns about potential zoonotic risks.

Challenges regarding the limitations of the use of both conventional and alternative control methods are also a cause for concern. Acaricides are not very effective against poultry red mite eggs, which makes reinfestation easier for mites [97]; plant-derived acaricides are only effective over short periods due to their volatile nature [63]; the use of natural predators and entomopathogenic microorganisms could be limited by environmental factors such as temperature and (for predators) alternative prey. The use of extreme temperatures can affect the integrity of the structures of poultry houses, while also being expensive to maintain; EU legislation requires a dark period of 8 h, making short light/dark period cycles impossible to achieve [2]. Inert substances have limited efficiency when used in highly humid environments, and they also cause irritation when inhaled by animals and workers [88]. And with integrated pest management, we must keep in mind the fact that this strategy, at its core, is a combination of the above-mentioned methods and is also heavily reliant on monitoring and evaluation, which is quite time- and labour-intensive. However, the biggest gap throughout all control methods might be the underestimation of preventive practices.

Given the challenges of existing methods, developing commercially viable vaccines is a priority. Vaccines could protect poultry without harming birds, leaving residues, or causing environmental damage, while also avoiding acaricide resistance [28]. However, vaccine development is complex and time-intensive, requiring the identification of effective candidate antigens. Even with advances in identifying antigens, progress remains slow [98,99]. Still, emerging technologies like next-generation sequencing, proteomics, and machine learning offer hope. These tools could streamline antigen identification, accelerating vaccine research and trials. Despite challenges, extensive research and innovative approaches may make effective vaccines a reality.

## 8. Genetic Research and Phylogenetic Insights

Genetic diversity and phylogenetic relationship studies are essential to understand the population dynamics of *Dermanyssus gallinae*, as well as to gain insight into its distribution. As previously mentioned, in the genus *Dermanyssus*, members of the *gallinae* group share quite similar traits, making them virtually impossible to distinguish from one another. Therefore, genetic diversity studies usually rely on markers for identifying intraspecific variations between PRM populations, such as the mitochondrial cytochrome c oxidise subunit I (COI) gene, the 16S rRNA gene, and the internal transcribed spacer (ITS) sequence, with the latter found to be the least useful [100]. Many genetic diversity studies based on these nucleotide sequences have been conducted in different parts of the world, including the USA, Brazil, Australia, Japan, and Europe, all of which suggest the intra- and international migrations of mites [101]. The presence of shared haplotypes in different countries basically underlines the previously mentioned fact that human activity, such as transport and trade routes or shared equipment, contributes to the distribution of mites. The genetic diversity of poultry red mites is highly varied depending on location, both in specific regions and between regions (Table 2). Generally speaking, when low genetic diversity cannot be explained by geographical factors (for example, neighbouring countries), the only possibilities are a shared origin of mites or frequent admixture through trade routes or sometimes even through mite transmission by wild birds [102,103]. On the other hand, greater genetic diversity suggests the opposite. A 2014 study from Italy showed significant genetic differences from other European countries, which suggested a lack of commercial transports and therefore mite exchanges [104]. In-region genetic diversity is also quite varied depending on the region of interest. For example, sequences from the UK showed greater diversity than sequences from Italy, which can be attributed to different levels of pressure for selection [105]. While it would be interesting to compare genetic diversity values with the favoured control methods from each country to determine which strategy exerts more selection pressure on poultry red mites, the variety of methods and the lack of concrete information about the frequency of their use in different countries make this difficult. However, with the help of questionnaires, for example, this comparison could be achievable in the future.

Genetic research is also an important part of understanding *D. gallinae*, and with the rise of NGS technology, this field has reached new heights. The first breakthrough came in 2014, when Schicht et al. [106] published the transcriptome dataset of *Dermanyssus gallinae*, after which, in 2018, Burgess et al. [107] released a draft genome assembly for PRMs. Both studies were crucial sources of genetic knowledge for bodies of research to come [106,107]. Since then, several genetic research papers have been published, largely focused on the identification of genes responsible for acaricide resistance. It has been revealed that *D. gallinae* achieves phoxim and cypermethrin resistance through target site insensitivity and the continuous overexpression of various detoxification enzymes and xenobiotic defence genes [90]. Scientists were also able to characterise such detoxification proteins, like glutathione-S-transferase (GST) and carboxylesterase (CarE) [108,109], further contributing to our understanding of poultry red mites.

It has been proved that genetic and phylogenetic studies are of fundamental importance. By being familiar with *D. gallinae*’s distribution networks, we can aim to eliminate them. By understanding the intricacies of resistance, we can try to circumvent them, and by continuous research, we can find new and effective ways for control. Genetic research itself could also be integrated into PRM management. An initial screening of mites from the infestation site could reveal their potential resistance, so that recommendations could be given for the choice in acaricides [110]. Eliminating the uncertainty of the chosen pesticide could also encourage confidence in the recommended one, which might result in farmers following the prescribed dosage, ultimately resulting in fewer resistant species.

## 9. Future Directions and Recommendations

*Dermanyssus gallinae* control requires innovative approaches as conventional acaricides become less effective and consumer demand for pesticide-free products increases. The development of vaccines against *D. gallinae* presents significant potential but faces several challenges. Current genomic data remain in draft form, falling behind similar arthropod initiatives. Completing high-quality genome assemblies is essential for supporting vaccine development and other control strategies [90,106,107]. While advances in genomic tools are accelerating antigen discovery, commercialization timelines remain extended, with proof-of-concept studies likely within 5 years and broader implementation potentially requiring a decade or more, depending on regulatory approval and cost effectiveness assessments.

While pursuing new technologies, current control methods should not be dismissed. Success in *D. gallinae* management requires a comprehensive approach comparable to building a house—prevention forms the foundation, effective alternative methods provide the structure, and conventional chemicals should be considered only after other options are explored. This systematic approach is exemplified in integrated pest management, which, despite higher initial costs and time investment, ultimately reduces losses while protecting animal welfare.

Research priorities for advancing *D. gallinae* control should focus on several key areas. Vaccine development efforts need to concentrate on identifying and validating protective antigens, developing effective delivery systems, and assessing cross-protection against different mite populations. Monitoring and detection systems require improvement through automation and early warning capabilities, supported by integrated data management platforms. Alternative control strategies deserve continued attention, including the optimisation of biological control agents, development of targeted attractant-based traps, and enhancement of physical control methods. Additionally, standardised economic impact assessments are needed to better understand the true cost of infestations and evaluate the cost effectiveness of various control methods.

The successful implementation of these research priorities requires strong coordination between researchers, industry stakeholders, and farmers. Knowledge transfer between these groups is crucial to ensure research findings translate into practical, field-applicable solutions. Furthermore, encouraging information sharing among stakeholders could help identify effective management practices and accelerate the adoption of successful control strategies [74,75,76]. Through these collaborative efforts and continued research focus, the challenges posed by *D. gallinae* can be better addressed, leading to more effective and sustainable control methods for the future.

## 10. Conclusions

*Dermanyssus gallinae* is perfectly adapted to the conditions of modern poultry farming. It can live comfortably in hen houses, where hiding places are in abundance and food is plenty. It can multiply its population in a matter of weeks, which, combined with the fact that it is able to starve for long periods, means that it can infest multiple flocks. *D. gallinae* has also adapted to the use of chemical compounds, referred to collectively as acaricides. With high prevalence in many countries on all continents, *Dermanyssus gallinae* is, without a doubt, the true plague of the poultry sector. Genetic and phylogenetic research was fundamental in understanding PRM and its distribution, while also shining a light on the importance of human activity as a factor of mite transmission. In the future, these types of studies from all PRM-affected countries could serve as a basis for recognising infested trade routes and eliminating mites that originate from other geographical regions. The next breakthrough in poultry red mite control would be the development of vaccines; however, vaccine development is a lengthy process. Algorithms utilising the processing power of computers could, in theory, help speed up the process of antigen identification; however, the best practices and algorithms are yet to be determined. Machine learning could also be utilised in early detection. Integrated pest management methods could be the best among available strategies; however, they also require a good understanding of current methods and their workings. Further research is urgently needed for determining the viability of algorithm use and also for sustainable management practices, with the former also requiring an open mind.

## Figures and Tables

**Table 2:** Genetic diversity of *Dermanyssus gallinae* in different European countries, offering recommendations for control formulated in studies based on [95].

Country	Genetic Diversity (H)	Justification
Belgium	0.964	High genetic diversity leads to increased resistance, necessitating the use of integrated pest management (IPM) to mitigate the problem.
United Kingdom	0.901	High genetic diversity. IPM and biological control measures should be used.
Albania	0.889	Due to high gene diversity, alternative methods should be considered to decrease the risk of resistance.
France	0.733	Given moderate diversity, potential biological methods warrant exploration.
Italy	0.722	Moderate diversity suggests a need for IPM to prevent long-term resistance.
Greece	0.521	Moderate diversity suggests less immediate pressure for alternative control methods.
Turkey	0.333	Low genetic diversity results in slower resistance development, suggesting that continued reliance on chemical controls should still prove effective.
Romania	0.286	Very low diversity implies a lower immediate risk of resistance, indicating that chemical controls remain effective.

## Data Availability

No new data were created or analysed in this study. Data sharing is not applicable to this article.
